# Effects of in-utero personal exposure to PM_2.5_ sources and components on birthweight

**DOI:** 10.1038/s41598-023-48920-w

**Published:** 2023-12-11

**Authors:** Karl O’Sharkey, Yan Xu, Jane Cabison, Marisela Rosales, Tingyu Yang, Thomas Chavez, Mark Johnson, Deborah Lerner, Nathana Lurvey, Claudia M. Toledo Corral, Shohreh F. Farzan, Theresa M. Bastain, Carrie V. Breton, Rima Habre

**Affiliations:** 1 Department of Population and Public Health Sciences, University of Southern California, 1845 N Soto St., Los Angeles, CA 90089 USA; 2 Spatial Sciences Institute, University of Southern California, Los Angeles, CA USA; 3 Department of Health Sciences, California State University Northridge, Northridge, CA USA; 4 Eisner Health, Los Angeles, CA USA

**Keywords:** Risk factors, Environmental chemistry, Epidemiology, Reproductive biology, Developmental biology, Diseases

## Abstract

In-utero exposure to fine particulate matter (PM_2.5_) and specific sources and components of PM_2.5_ have been linked with lower birthweight. However, previous results have been mixed, likely due to heterogeneity in sources impacting PM_2.5_ and due to measurement error from using ambient data. Therefore, we investigated the effect of PM_2.5_ sources and their high-loading components on birthweight using data from 198 women in the 3rd trimester from the MADRES cohort 48-h personal PM_2.5_ exposure monitoring sub-study. The mass contributions of six major sources of personal PM_2.5_ exposure were estimated for 198 pregnant women in the 3rd trimester using the EPA Positive Matrix Factorization v5.0 model, along with their 17 high-loading chemical components using optical carbon and X-ray fluorescence approaches. Single- and multi-pollutant linear regressions evaluated the association between personal PM_2.5_ sources/components and birthweight, adjusting for gestational age, maternal age, race, infant sex, parity, diabetes status, temperature, maternal education, and smoking history. Participants were predominately Hispanic (81%), with a mean (SD) gestational age of 39.1 (1.5) weeks and age of 28.2 (6.0) years. Mean birthweight was 3295.8 g (484.1) and mean PM_2.5_ exposure was 21.3 (14.4) µg/m^3^. A 1 SD increase in the mass contribution of the fresh sea salt source was associated with a 99.2 g decrease in birthweight (95% CI − 197.7, − 0.6), and aged sea salt was associated with a 70.1 g decrease in birthweight (95% CI − 141.7, 1.4). Magnesium, sodium, and chlorine were associated with lower birthweight, which remained after adjusting for PM_2.5_ mass. This study found evidence that major sources of personal PM_2.5_ including fresh and aged sea salt were negatively associated with birthweight, with the strongest effect on birthweight from Na and Mg. The effect of crustal and fuel oil sources differed by infant sex with negative associations seen in boys compared to positive associations in girls.

## Introduction

Low birthweight (LBW) is an endemic negative health outcome, with an estimated 8.3% of newborns born in the United States (U.S.) having a birthweight below 2500 grams (g)^1^. LBW is known to be associated with several negative health outcomes, including infant mortality^2,3^, later-life obesity^4^, diabetes^5^, cardiovascular disease^6,7^, and impaired-cognitive development^8,9^. Many of these negative health outcomes often disproportionately affect race/ethnicity groups; for example, obesity and type-2 diabetes prevalence are highest in Hispanic and Black communities^10,11^.

Combined with the greater burden of some negative health outcomes faced by Hispanic and Black communities, they also experience the greatest cumulative burden of air pollution exposure^12,13^. Various epidemiological studies, including several meta-analyses across the world, have found a modest association between ambient air pollution exposure during the in-utero period and birthweight, including LBW^14–17^. Of these ubiquitous air pollutants, a moderate association between particulate matter (PM) with an aerodynamic diameter less than 2.5 µm (PM_2.5_) and lower birthweight has been found using both ambient^18–20^ and personal monitoring approaches for PM_2.5_ exposure assessment^21,22^. Exposure to PM_2.5_ likely creates a hostile intrauterine environment which is hypothesized to explain its toxic effects, and while the biological mechanisms behind this effect are still emerging, studies suggest that oxidative stress, DNA methylation, and endocrine disruption may be at play and may lead to placental inflammation and growth restriction^23,24^. A recent meta-analysis highlighted the 3rd trimester to be an important window of vulnerability for the effect of ambient PM_2.5_ on birthweight^15^, possibly due to this being the period where most fetal growth occurs and therefore, particularly susceptible to biological disruption^25^. This may be due to effects on the maternal inflammatory pathways in late pregnancy, which contribute to the risk of adverse pregnancy outcomes^26^.

However, while reviews have concluded that there is a relationship between exposure to ambient PM_2.5_ and decreases in birthweight^15,17,27^, there is a great deal of heterogeneity in the findings, possibly due to measurement error from estimating personal exposure to PM_2.5_ of outdoor origin from ambient data^28,29^. Additionally, this may also be explained in part by the fact that PM_2.5_ is actually a mixture of organic and inorganic chemicals, with specific sources and/or components of PM_2.5_ being shown to differ in their toxicity with respect to birthweight^12,30,31^.

In our prior study using personal monitoring in the 3rd trimester, there was not a pronounced association between total personal exposure to PM_2.5_ and birthweight; however, specific indoor sources (such as candle and incense smoke) and exposure to PM_2.5_ more impacted by sources of outdoor origin appeared to be more strongly associated with lower birthweight^32^. These earlier findings are in line with the literature, with studies showing exposure to traffic-related sources including on-road gasoline and diesel traffic were negatively associated with birthweight^30,33,34^. Also, secondhand smoke (SHS) exposure is associated with reduced birthweight and increased risk of LBW^35,36^. To date, it is unclear whether the risk associated with these sources may be driven by exposure to the source itself as a unique mixture of pollutants or by any of its specific marker or signature chemical components. Therefore, in this analysis, we propose to investigate the effects of both major contributing sources of personal PM_2.5_ and their high-loading or signature components on birthweight.

This is an important question since the chemical composition of PM_2.5_ in conjunction with other properties like size distribution determines particles’ toxicity. The chemical composition of PM_2.5_ exposures may also differ by race/ethnicity with researchers finding that Hispanic individuals are exposed to elevated levels of 13 out of 14 PM_2.5_ components they investigated compared to non-Hispanic Whites in California^33^. Of these components, several were linked to increased risk of LBW and reduced birthweight^27,33^. For example, Basu et al.^33^ found significant reductions in birthweight per 1 IQR increase in exposure to outdoor vanadium ($\beta :$ − 32; 95% CI − 38, − 27), titanium ($\beta$: − 15; 95% CI − 17, − 13), zinc ($\beta$:-10; 95% CI − 12, − 7), and elemental carbon ($\beta$:-16; 95% CI − 19, − 14)^33^. Furthermore, a meta-analysis from Sun et al.^27^, corroborated these findings, but they also found that other components including silicon and nickel were elevated in Hispanic neighborhoods compared to Non-Hispanic White neighborhoods and were also negatively associated with birthweight.

However, as with the existing health literature on the effects of PM_2.5_ mass, there is a great deal of heterogeneity of results when looking at the mixture of sources and components that compose it^12,27,37^. One possibility is that individual exposure is assigned using estimates of outdoor concentrations at the residential level^38^, which fail to account for time-activity patterns and infiltration of outdoor pollutants indoors^29,39^, which introduces exposure measurement error. However, this error might be exacerbated when investigating the effects of chemical components of PM_2.5_ because of their greater spatial variability relative to PM_2.5_ mass concentration as a whole^40^. Additionally, total personal exposure to major contributing sources and components of PM_2.5_ also includes contributions from indoor sources and personal activity or behavior-related sources and not just outdoor sources.

Therefore, in this analysis, we aimed to investigate the relationship between exposure to six chemically derived major sources of personal PM_2.5_ in the 3rd trimester of pregnancy with infant birthweight. To accomplish this goal, we leveraged personal measurements of exposure to PM_2.5_ mass and its components and source apportionment models. We fit single and multi-pollutant models for the sources and also as a secondary aim, we investigated the independent effects of their high-loading “signature” chemical components to determine whether particular elements were driving the adverse health effects of the source mixtures.

## Materials and methods

### Study population

This work takes place in a 214-participant personal PM_2.5_ exposure monitoring sub-study nested within the Maternal and Developmental Risks from Environmental and Social Stressors (MADRES) study, an ongoing prospective cohort study of just over 1000 pregnant, primarily Hispanic, low-income pregnant women in Los Angeles County^41^. MADRES aims to investigate the cumulative impact of environmental pollutants and psychosocial, behavioral, and built environmental risk factors on maternal and infant health outcomes as described in more detail elsewhere^41^. Briefly, pregnant women were enrolled in the cohort through partnerships with four prenatal care clinics in Los Angeles, CA from November 2015. Eligibility for this study included: (1) at least 18 years old, (2) a singleton birth, (3) less than 30 weeks gestation at recruitment, (4) HIV negative, (5) having no physical, mental, or cognitive disability that would prevent the participant from providing informed consent, and (6) not currently incarcerated.

Of the 214 participants in the personal exposure monitoring study, nine were removed due to incomplete or erroneous personal PM_2.5_ mass exposure data or birth outcome data. Four participants did not have PM_2.5_ source data and were removed from the analysis. A multivariate k-nearest neighbor outlier detection analysis revealed three outliers in terms of personal exposure to the six sources. These were excluded from further analysis. However, given these points were very influential in the models, results including and excluding them are presented side-by-side for completeness in this analysis. This resulted in a sample of 198 mother-infant dyads used in the final models (201 in the outlier-included models).

Participants were recruited by trained, bilingual MADRES staff members during a 3rd trimester visit to the University of Southern California (USC) clinic, where consenting women were asked to participate in the in-utero personal exposure monitoring sub-study for a 48-h monitoring period. This sample was comparable to the larger MADRES cohort on key demographics, birth outcomes, and ambient air pollution metrics.

### Personal PM_2.5_ exposure monitoring

Total personal PM_2.5_ exposure was measured over an integrated 48-h monitoring period in the 3rd trimester using a custom-designed sampling protocol between October 2016 and February 2020. The 3rd trimester was chosen because most fetal weight gain occurs in this trimester^25^. Participants were shown and provided with instructions by trained staff members on the correct use of the personal exposure monitoring device, which was housed in a crossbody purse. Instructions included a demonstration of how to wear the purse, making sure to keep the sampling inlet located on the purse shoulder strap free from obstructions and in the breathing zone. Additionally, participants were requested to wear the device as much as possible during normal daily activities, with a limited number of exceptions, including driving, showering, sleeping, etc. Participants were asked to keep the sampling device safe and away from water, high humidity (such as showering), heat, children, and pets, and when unable to wear the device, place it as near as possible, such as on the passenger seat if driving, and a side-table while sleeping.

The purse contained a Gilian Plus Datalogging Pump (Sensidyne Inc.) connected to a Harvard PM_2.5_ Personal Environmental Monitor (PEM) with a pre-weighed 37 mm Pall Teflo filter. The device was programmed to start at midnight the day after enrollment into the sub-study, and actively sampled at a 50% cycle and a 1.8 L per minute (LPM) flow rate. The sampling device was programmed to shut off after the 48-h sampling period and collected by staff members the following day when a brief exit survey was conducted. The devices were then transferred to the USC Exposure Analytics lab for analysis. Pump data was downloaded, evaluated for errors, and stored securely. Filters were equilibrated within a dedicated chamber and gravimetrically weighed in temperature and relative humidity-controlled glove box using an MT-5 microbalance (Mettler Toledo, Inc.) to obtain PM_2.5_ mass concentration reported in μg/m^3^. The methodology of this personal monitoring study has been described in greater detail elsewhere^42^.

### Elemental speciation analysis using X-ray fluorescence

Elemental data was obtained via an X-ray fluorescence analysis^43^ that determined the elemental composition of PM_2.5_ collected on personal sampling filters. Concentrations of elements (reported in ng/m^3^) identified in the source apportionment analysis^44^ as markers or high-loading species in the source profiles were used in this current analysis. These included: aluminum (Al), barium (Ba), bromine (Br), calcium (Ca), chlorine (Cl), copper (Cu), magnesium (Mg), nickel (Ni), silicon (Si), sodium (Na), sulfur (S), titanium (Ti), vanadium (V), and zinc (Zn).

### Optical carbon fractions analysis

A multiwavelength optical absorption approach was used to measure concentrations of several carbon fractions (reported in μg/m^3^) in the personal PM_2.5_ samples, including: (1) Black Carbon (BC), (2) Brown Carbon (BrC), and (3) Environmental Tobacco Smoke (ETS). This method is described in more detail elsewhere^45^, and its performance is consistent with other carbon apportionment approaches^45^. Briefly, this method uses an integrating sphere radiometer which measures the difference in absorption of transmitted light passed through a mass-loaded Teflo filter. Each of the three carbon components measured with this approach has a different optical density at varying wavelengths, which allows for the identification and quantification of their respective concentration from their optical properties. For the purposes of this study, ETS refers to the carbon fraction concentration obtained via this multiwavelength optical analysis, while the secondhand smoke (SHS) source (explained below) refers to one of the six major contributing sources of personal PM_2.5_ identified in the PMF analysis. This source had high loadings of several different but highly correlated components, namely ETS and BrC.

### Personal PM_2.5_ sources

Six major contributing sources of personal PM_2.5_ were used in this analysis, obtained from an earlier source apportionment analysis of these personal exposure filter samples using the EPA Positive Matrix Factorization model (EPA PMF v5.0)^44^. The PMF analysis used PM_2.5_ mass and the concentrations of 36 components (33 elements and 3 optical carbon fractions) as inputs to derive the six sources and their predicted mass contributions. The elements and carbon species were obtained from X-ray fluorescence (XRF) and multiwavelength optical absorption carbon speciation analyses, respectively, at the Research Triangle Institute International, Inc (described in more detail below). For this current study, only 17 (14 elements and 3 optical carbon fractions) high-loading components or signature tracers of the six sources (noted in parentheses) were investigated with birthweight including:

(1) Traffic (BC, Zn, Ba), (2) Secondhand Smoke (BrC, ETS, Br), (3) Aged Sea Salt (S, Na, Mg), (4) Fresh Sea Salt (Cl, Na, Mg), Fuel oil (Cu, Ni, V), and Crustal (Si, Ca, Ti, Al).

### Birthweight outcome

Infant birthweight (grams) was abstracted from participants’ electronic medical records (EMR). Given that birthweight and gestational age are closely linked, birthweight-for-gestational age z-scores that were either sex or parity specific were also assessed, as described in^46^. However, the results were not materially different from continuous birth; therefore, only the continuous birthweight models are presented.

### Covariate data

Possible covariates were determined a priori from the air pollution and birth outcomes literature. Factors assessed included maternal demographics, pregnancy and birth outcomes, study design characteristics (such as hospital of birth), and meteorology. Self-report data were collected during the MADRES cohort follow-up through a sequence of staff administered in-person and telephone-based questionnaires. All questionnaires were available in either English or Spanish. Anthropometric assessments were conducted via regular clinic visits. Due to the timing of this personal monitoring study in the 3rd trimester of pregnancy, data primarily came from the 3rd trimester visit, the personal monitoring study exit survey, and birth-related datasets and variables, with additional variables assessed at the baseline visit including race/ethnicity and pre-pregnancy body mass index (BMI; kg/m^2^).

Additional pregnancy and birth-related covariates were also evaluated for confounding. Gestational age at birth (GA; weeks) was estimated with a hierarchical approach of differing methods from the preferred ultrasound measurement of crown-rump length at < 14 weeks gestation (60%), ultrasound measurement of fetal biparietal diameter at < 28 weeks’ gestation (30%), and from physicians’ clinical estimate from EMR (10%). Parity was defined as 1 or more previous births and included a missing category with 6 participants so as not to lose sample size. Infant sex was obtained through EMR.

Maternal demographics included the following: Age at baseline (continuous; years), education (completed < 12th grade, completed high school, at least some college), household income (less than $15,000, $15,000–29,999, $30,000+, don’t know), and diabetes status [no diabetes, glucose intolerant, diabetes (chronic and gestational)]. Race/ethnicity was categorized as Hispanic, non-Hispanic Black, and non-Hispanic Other. Pre-pregnancy BMI (continuous; kg/m^2^) was calculated from self-reported pre-pregnancy weight and standing height measured by MADRES staff at participants’ first visit by either a stadiometer (Perspectives model PE-AIM-101) or EMR. Self-report weight was chosen because participants entered the study at differing weeks of gestation.

Meteorological factors evaluated in this study included ambient air temperature (Celsius) (calculated as the average of minimum and maximum air temperature) and relative humidity (%), averaged over the 3rd trimester and estimated at the residential location based on a high-resolution (4 km × 4 km) gridded surface meteorological dataset ^47^, and seasonality using the following levels, “Cool (Winter), Warm (Summer), and Transition (Spring and Autumn)”.

### Statistical analysis

#### Descriptive statistics

Descriptive statistics of key sample demographics and mean and standard deviations were calculated for concentrations of personal PM_2.5_ mass, six PMF-derived sources of personal PM_2.5_, and 17 high-loading components. The distribution of birthweight, personal PM_2.5_ mass concentration, and each source and component were investigated to identify any issues with normality and potential influential points. Bivariate analyses using Kruskal–Wallis one-way analysis of variance tests and Spearman’s correlation coefficients were conducted between personal PM_2.5_ mass, its major contributing sources, and birthweight by various sample characteristics to elicit any additional potential confounders for our regression analysis, in addition to those identified in previous literature^27^.

#### Linear regression models

Single- and multi-pollutant linear regression models were used to investigate the primary aim of this study, that is, the association between in-utero exposure to major personal PM_2.5_ sources and birthweight, adjusting for gestational age at birth, maternal age, race/ethnicity, infant sex, parity, diabetes status, temperature, maternal education, and personal smoking history. Even though this study assessed SHS as a source of PM_2.5_, it did not correlate strongly with our smoking covariate (never/ever smoker). However, this smoking covariate did seem to be a confounder and impact our main effects, therefore, it was kept within the model. Meteorological variables, such as season, were excluded from the models after statistical confounding checks showed no material differences in the main effects. This was done to conserve statistical power. The effect of total personal PM_2.5_ on birthweight, previously reported by this group^32^, was included in relevant tables for comparison purposes. PM_2.5_ sources that were not highly correlated with one another, as determined by a bivariate Spearman correlation analysis and/or a variance inflation factor (VIF) below 10 in the regression, were used to evaluate the effect of each source on birthweight, adjusting for one or more other PM_2.5_ sources. Multi-pollutant models were conducted with up to four personal PM_2.5_ sources included in each model; however, three- and four-pollutant model results did not materially differ. Therefore, only single- and two-pollutant models are reported. Additionally, the association between PM_2.5_ and birthweight has been shown to differ by the sex of the infant, therefore, this study evaluated whether the effect of each PM_2.5_ source exposure on birthweight was modified by sex. Non-linear effects were evaluated by modeling each PM_2.5_ source on birthweight using generalized additive models (GAMs) and assessing Akaike information criterion (AICs) vs. linear regression models. As a sensitivity analysis, the association of each PM_2.5_ source on birthweight was examined only among full-term births (37 weeks or older gestation) due to apprehensions about potential bias from factoring in gestational age as a confounder, given the possibility that it might act as a mediator^48^. This was conducted with and without the inclusion of gestational age as a covariate.

For the secondary aim of this study, this study attempted to determine whether it was the source itself (a particular mix of components) driving any adverse health effects or an individual element or component. To evaluate whether it is the PM_2.5_ source (the mixture) or any of its high-loading components that are driving the observed association between sources and birthweight, the effect of the 17 high-loading PM_2.5_ components on birthweight was investigated. Additionally, because PM_2.5_ mass concentration may be related to both birthweight and the concentration of the PM_2.5_ components (especially more abundant ones), further analyses adjusting component models for PM_2.5_ mass were performed via two approaches, which have different interpretations, while similarly attempting to account for PM_2.5_ mass itself. The first was by adjusting for PM_2.5_ mass in the individual component models by directly including it as a simple covariate or potential confounder. According to Mostofsky et al.^49^, this parameter “represents the impact of higher levels of the constituent (and its correlates), holding the other constituents constant”^49^. The second approach was to create component residuals by regressing the mass concentration of each PM_2.5_ component (dependent) on the total PM_2.5_ mass concentration (independent). The component coefficient “represents the increase in risk associated with higher levels of the constituent while holding PM_2.5_ constant”^49^.

Due to concerns with outliers being influential as determined by model diagnostics in 3 out of 6 main source models, a multivariate K-nearest neighbor outlier detection analysis was conducted in JMP Pro 16 (SAS Institute, Inc., Cary, NC, USA). This was used to identify outliers up to a distance of 8 nearest neighbors along the concentrations of all six personal PM_2.5_ sources. This analysis allowed us to objectively identify data points that were materially different from the overall sample across six dimensions. All effect estimates and 95% confidence intervals were scaled and reported per 1 SD increase in the main exposure of interest. An alpha of 0.05 was selected as a priori significance level for our main exposure/outcome analyses, while 0.10 was used for the infant sex interaction analyses to allow more leniency for statistical interactions due to the increased power requirement. Model diagnostics were conducted to ensure models were not affected by multicollinearity or influential points. The analysis was conducted using SAS v9.4 (SAS Institute, Inc., Cary, NC, USA).

### Ethical approval and consent to participate

Study procedures were approved by the USC Institutional Review Board (IRB) and all participants completed written informed consent at the first study visit (IRB: HS-16-00530). This study was performed according to the ethical guidelines expressed in the Strengthening of the Reporting of Observational Studies in Epidemiology (STORB) guideline and the Declaration of Helsinki.

## Results

### Descriptive statistics

Sample characteristics for the full sample are presented in Table 1 The participants of this study were predominantly Hispanic (81%), lower income (43% had income below $30,000), with a mean age of 28 years, and 63% had a previous pregnancy. Around 70% of the women were overweight or obese, and 34% had glucose intolerance or diabetes (chronic or gestational). Participants’ infants were 51% female and had a mean (SD) birthweight of 3295.8 (484.1) grams and gestational age of 39.1 (1.5) weeks at the time of birth.

**Table 1:** Sample participant characteristics (N = 201).

Variable	Mean (SD) or n (%)	Variable	Mean (SD) or n (%)
Birthweight (g)	3295.8 (484.1)	Pre-pregnancy BMI (kg/m^2^)	28.9 (6.8)
Maternal age (years)	28.2 (6.0)	Normal	61 (30.4%)
Gestational Age at Birth (weeks)	39.1 (1.5)	Overweight	61 (30.4%)
Sex		Obese	79 (39.3%)
Female	103 (51.2%)	Parity	
Male	98 (48.8%)	No	68 (33.8%)
Race/ethnicity		Yes	127 (63.2%)
Hispanic	163 (81.1%)	Missing	6 (3.0%)
Black, Non-Hispanic	22 (11.0%)	Maternal income	
Other, Non-Hispanic	16 (8.0%)	Less than $15,000	41 (20.4%)
Education		$15,000–$29,999	45 (22.4%)
< 12th grade	48 (23.9%)	$30,000+	42 (20.9%)
Completed High School	65 (32.3%)	Don’t know	73 (36.3%)
Some college +	88 (43.8%)	Smoking history	
Diabetes		Never	160 (79.6%)
Normal	133 (66.2%)	Ever	41 (20.4%)
Glucose Intolerant	45 (22.4%)	Outdoor Temperature (°C)	19.02 (3.5)
Diabetes^	23 (11.4%)		

BMI, body mass index; ^diabetes = chronic or gestational diabetes.

Total personal PM_2.5_ exposure had a mean (SD) of 21.3 (14.4) μg/m^3^. The average estimated mass contributions of the six personal PM_2.5_ sources were as follows: SHS 12.0 (9.2) μg/m^3^, crustal 2.1 (3.5) μg/m^3^, fuel oil 2.1 (1.6) μg/m^3^, aged sea salt 0.8 (0.8) μg/m^3^, fresh sea salt 0.9 (2.1) μg/m^3^, and traffic 0.5 (0.6) μg/m^3^ (Table 2). Carbon fractions concentrations were generally similar; however, ETS was the highest with a mean of 1.4 μg/m^3^ but also had the highest variability with a SD of 5.5 μg/m^3^. Sulfur, sodium, silicon, and chlorine were the elements measured at the highest concentrations, with a mean (SD) of 397.7 (283.9), 311.1 (305.7), 165.7 (200.0), and 129.5 (259.4) ng/m^3^, respectively (Table 2).

**Table 2:** Summary statistics of PM_2.5_ sources and components concentrations (N = 201).

Pollutant	Mean	SD	Pollutant	Mean	SD
Personal PM_2.5_ mass (μg/m^3^)	21.3	14.4	Elements (ng/m^3^)		
			Aluminum (Al)	12.3	48.0
Sources (μg/m^3^)			Barium (Ba)	14.6	13.7
Secondhand smoke (SHS)	12.0	9.2	Bromine (Br)	3.0	3.2
Crustal	2.1	3.5	Calcium (Ca)	85.8	143.2
Fuel oil	2.1	1.6	Chlorine (Cl)	129.5	259.4
Aged sea salt	0.8	0.8	Copper (Cu)	18.7	12.2
Fresh sea salt	0.9	2.1	Magnesium (Mg)	40.0	63.9
Traffic	0.5	0.6	Nickel (Ni)	2.3	2.7
			Sodium (Na)	311.1	305.7
Optical carbon fractions (μg/m^3^)			Silicon (Si)	165.7	200.0
Black carbon (BC)	1.0	1.5	Sulfur (S)	397.7	283.9
Brown carbon (BrC)	1.1	0.8	Titanium (Ti)	10.0	11.9
Environmental tobacco smoke (ETS)	1.4	5.5	Vanadium (V)	0.6	1.2
			Zinc (Zn)	13.2	16.9

PM_2.5_ = particulate matter with an aerodynamic diabetes less than 2.5 µm.

The relationships between personal PM_2.5_ mass and its six sources along with key demographics are presented in Table S.1 of the supplement. There was no noticeable difference in personal PM_2.5_ mass exposure by sociodemographic and other covariates. However, personal PM_2.5_ mass concentration was highest in Hispanic and non-Hispanic Black participants compared to non-Hispanic Others, and was roughly 2 and 4 μg/m^3^ greater in participants with diabetes (chronic and gestational) than those with glucose intolerance and without diabetes. Traffic, SHS, and Aged and Fresh Sea Salt were highest in Hispanic participants, crustal was highest in participants that did not complete high school, and SHS was highest in participants with glucose intolerance and those reporting < $15,000 maternal income.

There were modest associations between several of the PM_2.5_ sources (Table 3). Traffic was positively correlated with crustal (ρ = 0.35; *p* < 0.001) and negatively correlated with fresh sea salt (ρ = − 0.22; *p* = 0.002) and fuel oil (ρ = − 0.17; *p* = 0.017). SHS was negatively correlated with aged sea salt (ρ = − 0.22; *p* = 0.002), and fresh sea salt (ρ = − 0.26; *p* < 0.001). A Spearman correlation matrix for the components is presented in Table S.2 Spearman correlations varied between − 0.58 for ETS and BC (*p* < 0.001) to 0.86 for Na and Mg (*p* < 0.001). The majority of correlations were positive.

**Table 3:** Spearman’s correlation coefficients for major contributing sources of personal PM_2.5_ (N = 201).

Variables	Traffic	Secondhand smoking	Aged sea salt	Fresh sea salt	Fuel oil	Crustal
Traffic	1.00					
Secondhand smoke	− 0.08	1.00				
Aged sea salt	− 0.04	− **0**.**22**	1.00			
Fresh sea salt	− **0**.**22**	− **0**.**26**	0.08	1.00		
Fuel oil	− **0**.**17**	− 0.00	− 0.08	0.00	1.00	
Crustal	**0**.**35**	0.02	− 0.11	− 0.10	**0.14**	1.00

Notes: PM_2.5_ = particulate matter with an aerodynamic diabetes less than 2.5 µm; bolded = *p* value < 0.05.

### Associations of personal PM_2.5_ and components with birthweight

Overall, in the fully adjusted final models, there was a small negative association between personal PM_2.5_ exposure and birthweight (β = − 33.5; 95% CI − 103.2, 36.1) per 1 SD increase pollutant. There was an average decrease of 99.2 g (95% CI − 197.7, − 0.6) in birthweight for the fresh sea salt source. This result remained after adjusting individually for fuel oil and crustal sources but became marginally significant after adjustment for aged sea salt. Aged sea salt was associated with a 70.1 g (95% CI − 141.7, 1.4) decrease in birthweight. This remained after further adjustments for fresh sea salt, traffic, fuel oil, and crustal sources (Table 4).

**Table 4:** Single- and two-pollutant associations between PM_2.5_ sources and birthweight.

Model	Final model (N = 198)			Outliers included (N = 201)		
	β	95% CI		β	95% CI	
Single-pollutant models						
Personal PM_2.5_ mass	− 33.5	− 103.2	36.1	42.1	− 15.6	99.9
Personal PM_2.5_ Sources						
Traffic	22.2	− 35.8	80.3	25.6	− 34.4	85.5
Secondhand smoke (SHS)	− 12.8	− 70.2	44.6	− 16.3	− 75.0	42.4
Aged sea salt	− 70.1	− 141.7	1.4	− 46.1	− 115.0	23.2
Fresh sea salt	− **99**.**2**	− **197**.**7**	− **0**.**6**	15.3	− 43.6	74.1
Fuel oil	16.0	− 40.2	72.3	25.2	− 32.6	83.0
Crustal	− 11.9	− 98.6	74.7	72.9	15.7	130.2
Two-pollutant models						
Traffic						
Adjusted for SHS	21.4	− 37.0	79.7	24.6	− 35.6	84.8
Adjusted for aged sea salt	19.9	− 37.8	77.6	23.1	− 36.9	83.1
Secondhand smoking						
Adjusted for fuel oil	− 12.6	− 70.1	44.9	− 16.2	− 75.0	42.6
Adjusted for traffic	− 11.2	− 68.8	46.5	− 14.8	− 73.7	44.1
Adjusted for crustal	− 12.6	− 70.1	45.0	− 19.8	− 77.8	38.1
Aged sea salt						
Adjusted for fresh sea salt	− 69.1	− 140.1	2.0	− 56.8	− 129.4	15.9
Adjusted for traffic	− 69.1	− 140.9	2.7	− 44.1	− 113.5	25.2
Adjusted for fuel oil	− 70.3	− 142.0	1.5	− 45.6	− 114.7	23.6
Adjusted for crustal	− 70.3	− 142.1	1.5	− 40.6	− 109.0	27.7
Fresh sea salt						
Adjusted for aged sea salt	− 97.7	− 195.6	0.1	30.1	− 30.5	92.7
Adjusted for fuel oil	− **100**.**4**	− **199**.**2**	− **1**.**6**	14.8	− 44.1	73.7
Adjusted for crustal	− **103**.**5**	− **203**.**4**	− **3**.**7**	19.4	− 38.7	77.5
Fuel oil						
Adjusted for aged sea salt	16.3	− 39.5	72.2	24.8	− 32.9	82.5
Adjusted for fresh sea salt	18.2	− 37.6	74.0	24.9	− 33.0	82.9
Adjusted for SHS	15.9	− 40.5	72.3	25.1	− 32.8	83.3
Crustal						
Adjusted for aged sea salt	− 12.9	− 98.9	73.1	70.8	13.5	128.1
Adjusted for SHS	− 11.5	− 98.4	75.4	73.9	16.5	131.2
Adjusted for fresh sea salt	− 25.4	− 112.3	61.4	74.0	16.6	131.4

PM_2.5_ = particulate matter with an aerodynamic diabetes less than 2.5 µm; SHS = secondhand smoke PM_2.5_ source; β = change in birthweight per 1 SD increase in pollutant; CI = confidence intervals; all models were adjusted for gestational age at birth, maternal age, race/ethnicity, infant sex, parity, diabetes status, temperature, maternal education, and personal smoking history.

**Figure 1:**
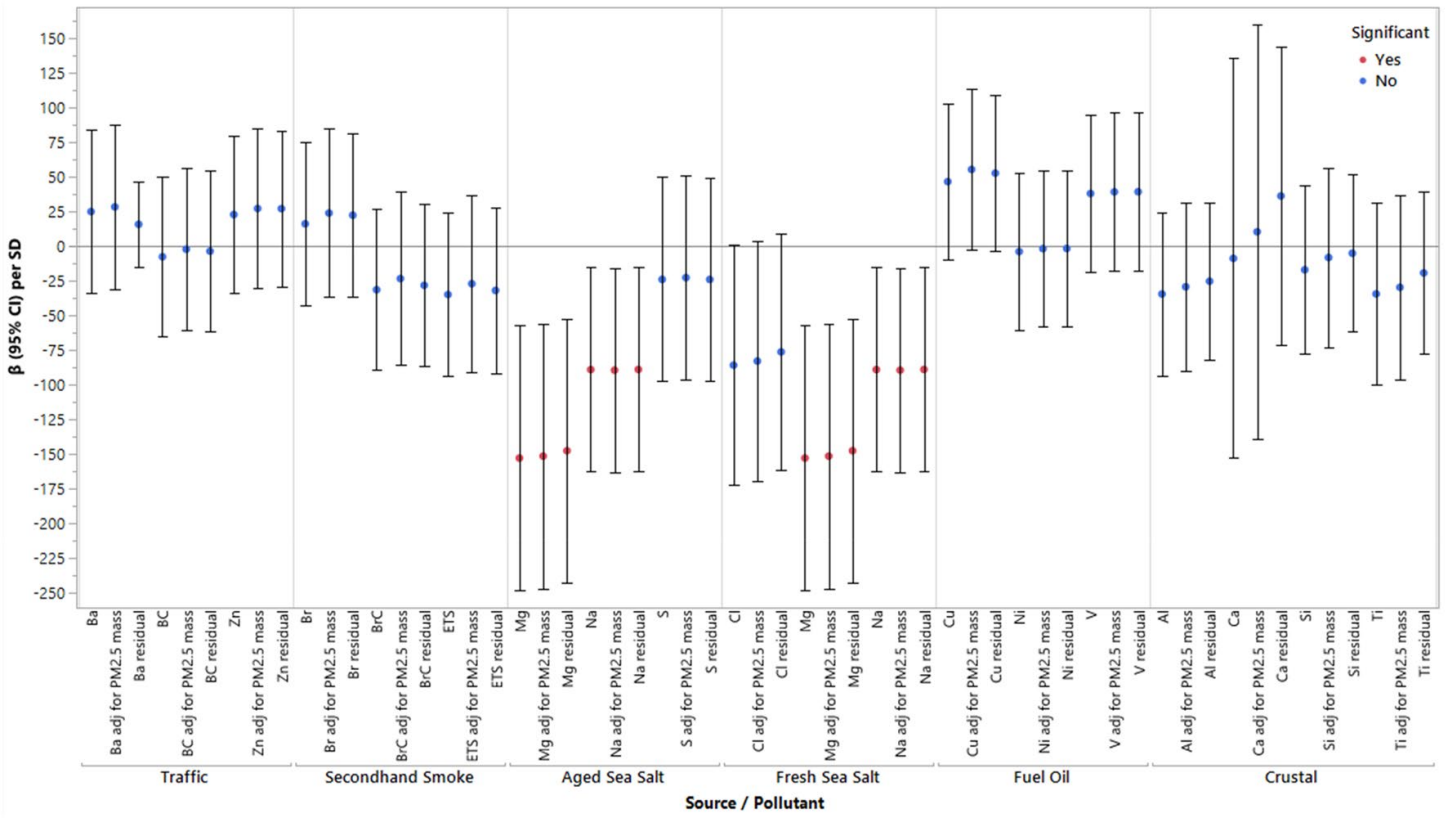
Associations Between High-Loading Components of the Six Personal PM_2.5_ Sources and Birthweight in Single-Pollutant Models, Adjusting for Personal PM_2.5_ Mass Concentration, and Using the Component Residuals as the Main Exposure, Respectively. *Notes*: PM_2.5_ = particulate matter with an aerodynamic diabetes less than 2.5 µm; significance < 0.05; β = change in birthweight per 1 SD increase in pollutant; adj = adjusting; all models were adjusted for gestational age at birth, maternal age, race/ethnicity, infant sex, parity, diabetes status, temperature, maternal education, and personal smoking history.

Two high-loading components of fresh sea salt and aged sea salt, Na (β = − 89.4; 95% CI − 163.0, − 15.7) and Mg (β = − 153.2; 95% CI − 248.7, − 57.7), were statistically associated with lower birthweight (Fig. 1). SHS as a source and two of the major contributing components had small negative associations with birthweight. The effect estimates did not materially change after adjustment for personal PM_2.5_ mass as a covariate or when looking at PM_2.5_ component residuals. In a sensitivity analysis of just full-term births, the results did not materially change when gestational age was removed as a covariate; however, there were slight differences when gestational age was also included in these models (Table S.3).

### Effects of personal PM2.5 sources on birthweight by infant sex

While not meeting statistical significance, the effect of personal PM_2.5_ on birthweight was more negative in males (β = − 63.3 g; 95% CI − 169.4, 42.8) compared to females (β = − 11.6 g; 95% CI − 103.0, 79.9; Table 5). The effect of the crustal source on birthweight was modified by infant sex, with males observing a − 83.4 g (95% CI − 187.9, 21.0) decrease in birthweight per 1 SD increase in exposure to the crustal source compared to a 127.0 g (95% CI − 17.4, 271.4) increase for females (Interaction: *p* = 0.020). Additionally, the effect of the fuel oil source on birthweight was − 31.1 (95% CI − 114.8, 52.5) for males and 55.0 (95% CI − 21.0, 130.9) for females (Interaction: *p* = 0.135).

**Table 5:** Estimated change in birthweight (g) per 1 SD increase in pollutant by infant sex (N = 198).

Variable	β	95% CI		*Interaction p* value
Interaction of personal PM_2.5_ mass by infant sex				0.464
Female	− 11.6	− 103.0	79.9	
Male	− 63.3	− 169.4	42.8	
Interaction of traffic by infant sex				0.785
Female	7.6	− 112.7	128.0	
Male	26.5	− 39.3	92.2	
Interaction of SHS by infant sex				0.934
Female	− 10.9	− 84.0	62.3	
Male	− 15.7	− 106.0	74.7	
Interaction of aged sea salt by infant sex				0.600
Female	− 82.9	− 169.0	3.3	
Male	− 51.5	− 151.6	48.5	
Interaction of fresh sea salt by infant sex				0.524
Female	− 138.1	− 293.6	17.5	
Male	− 73.7	− 199.9	52.6	
Interaction of fuel oil by infant sex				0.135
Female	55.0	− 21.0	130.9	
Male	− 31.1	− 114.8	52.5	
Interaction of crustal by infant sex				**0**.**020**
Female	127.0	− 17.4	271.4	
Male	− 83.4	− 187.9	21.0	

All models are adjusted for gestational age at birth, maternal age, race/ethnicity, infant sex, parity, diabetes status, temperature, maternal education, and personal smoking history; SHS, secondhand smoke source.

## Discussion

This analysis investigated the effect of major contributing sources of personal PM_2.5_ exposure on birthweight in the 3rd trimester within the MADRES in-utero personal exposure monitoring study. To our knowledge, this is the first analysis relating chemically derived sources of personal PM_2.5_ exposure with birthweight in a largely lower income, Hispanic, health disparities population leveraging “gold standard” personal exposure monitoring data. Prior studies reported concerns about exposure misclassification due to spatial variability of PM_2.5_ components when using ambient monitoring data to assign individual exposure^40^. This present study used sources derived from personal exposure monitoring data, which may remove much of this concern by measuring PM_2.5_ components for each individual in their personal breathing zone^39^.

Overall, this study found that total personal PM_2.5_ was marginally negatively associated with birthweight; however, some of its more specific sources, including fresh sea salt and aged sea salt were more strongly negatively associated with birthweight. Both sources contained Na and Mg as high-loading components, which were the elements that were most negatively associated with birthweight in this analysis. The findings in this present study differ from prior studies which found no association between sea or marine salt sources and reduced birthweight^50,51^. There are several possible explanations for the different findings. For example, Bell et al.^50^ was conducted in Connecticut and Massachusetts, while the present study was in California, likely resulting in regional differences in the mixture of marine or sea salt sources or other pollutants that track along with it. Additionally, differences in the timing of exposure assessment may also explain differences in results. Prior studies looking at the effects of outdoor Na concentrations on birthweight are limited; however, Basu et al.^33^ found that Na was associated with decreased birthweight in California. Similarly, the outdoor concentration of sodium ion (Na^+^) was associated with lower birthweight on the East Coast^37^. The relationship between Mg has been less studied, but a study in California found no association between Mg and birthweight^52^. It is not apparent why sea salt, particularly fresh sea salt, has the most adverse effect on birthweight in our study. It is possible fresh sea salt may be correlated with offshore marine shipping emissions given they both originate over the ocean and may be transported to receptor locations under similar meteorological and wind conditions. Components of marine shipping emissions (Ni, V, and EC) have been associated with lower birthweight^12,53^. However, in our study, the fuel oil personal exposure source (with loadings of Ni and V) was poorly correlated with fresh sea salt. Fresh sea salt as an outdoor source of PM_2.5_ is also correlated with being closer to the coast and the ports in Los Angeles, CA^54^, yet this spatial pattern was not apparent in our personal monitoring study for the fresh sea salt or the fuel oil sources. The fuel oil personal exposure source in our study may be capturing impacts of heavy-duty machinery and industrial equipment that burn heavier residual fuel oil, which is very common in Los Angeles, CA rather than picking up shipping emission signals from the ports^55^.

Fresh and aged sea salt did differ in an important way, with fresh sea salt containing higher loadings of Cl which is replaced with S as fresh sea salt undergoes photochemical reactions and becomes aged sea salt^56,57^. While the two sources share Na and Mg as high-loading components, they are not highly correlated. Differences in their impacts on birthweight may be due to other components or factors that correlate with them. For example, in our study, the effect of Cl on birthweight was stronger than S per 1SD of each pollutant. Previously, S was associated with decreased birthweight^33,58^, while results for Cl have been mixed with two studies finding reductions in birthweight^33,37^, while another found no association with LBW risk^59^. Interestingly, these studies found an association with Cl and decreased birthweight in New England and California, highlighting that this may not be a local phenomenon.

Other considerations for why aged sea salt might be negatively associated with birthweight are through secondary formation processes. Aged sea salt as a personal exposure source was highly correlated with outdoor ozone and temperature in our study. Both ozone and aged sea salt undergo chemical aging and transformation processes in the atmosphere under similar conditions of high temperature which could explain this correlation^54,57,60^. However, 8-h maximum ozone concentration was not associated with birthweight for the same 48 h sampling period or the whole third trimester within our sample (estimated at the residence using inverse distance weighted squared spatial interpolation, data not shown). This suggests there may be other processes, factors, or co-exposures associated with personal exposure to fresh and aged sea salt—both of clear outdoor origin—that may also be negatively associated with birthweight.

When components are more negatively associated than the source itself, it may imply that that particular compound is more toxic than the whole mixture. Efforts to identify sources or important components can lead to actionable interventions. For example, researchers found that stricter caps in Europe on sulfur content in marine fuel led to 22% reductions in sulfur dioxide gas and 6% reductions in PM_2.5_, which in turn resulted in a 7% reduced risk of LBW^61^. Similar policies have been implemented in California^62^. Additionally, in California, while there has been a general reduction in ambient PM_2.5_ concentrations over the past 20 + years due to regulatory interventions^63^, this study adds to the literature that specific sources or components of PM_2.5_ still place pregnant mothers at risk of adverse birth outcomes.

This study found that SHS and two high-loading components (ETS and BrC) were negatively associated with birthweight; however, the effect estimates for ETS and BrC were more negative than the SHS source itself. Overall, our results agree with prior studies that found a negative association between SHS and birthweight^36,64^. Additionally, a prior study investigating the effect of total personal PM_2.5_ on birthweight, found that self-reported prenatal SHS reduced birthweight^22^.

This current study did not find a particularly strong association between the personal traffic exposure source or its components. This differs from prior studies that have generally concluded that traffic-related exposures are related to lower birthweight^65^ and an increased risk of LBW^51–53^. Personal exposure to Zn was not associated with lower birthweight, which is inconsistent with the consensus of prior studies of ambient Zn^27,33^, although others have also found no association^30^. Zn is correlated with tire wear, one of the two dominant non-tailpipe emissions^66^. The personal traffic exposure source used in this study is characterized by high loadings of BC which is a marker of tailpipe combustion but also contains high loadings of Ba and Zn, markers of non-tailpipe or non-exhaust brake and tire wear emissions, respectively. As such, the traffic source may be capturing both fuel combustion and abrasive vehicular wear emissions combined^54^. Interestingly, while researchers in London found an increased risk of LBW from non-exhaust traffic ambient PM_2.5_ (did not hold up after adjustment for other air pollutants), the risk of LBW associated with non-exhaust traffic ambient PM_2.5_ was consistently lower across all models compared to traffic-exhaust related ambient PM_2.5_^67^. Additionally, different spatial scales may explain the differences observed between studies as prior literature has used ambient monitoring compared to personal monitoring in this study. Similarly, personal exposure to the fuel oil source was not associated with reductions in birthweight in this study; however, there was evidence that infant sex may modify this relationship.

This study found a small negative association between crustal exposure and lower birthweight. Additionally, a statistical interaction was found by infant sex, with negative effects seen in males and positive effects in females. Together with fuel oil, this provides evidence for potential differences in the underlying biomechanisms of how air pollution affects health, which may interact with sex-based biological differences among fetuses. Notably, unlike other components we investigated, effect estimates for crustal components were the most altered when adjusted for personal PM_2.5_ mass. This suggests that the effect of these components may be confounded by the effect of PM_2.5_ mass because PM_2.5_ may be correlated with both the component and the health outcome^49^, possibly because crustal components are more abundant and therefore more correlated with PM_2.5_ mass.

This study has several strengths, including the use of chemical speciation data and source apportionment derived PM_2.5_ sources, from “gold standard” personal exposure monitoring data. Prior studies that assessed PM_2.5_ sources and components^27,30^ used outdoor measurements which do not account for exposures that occur indoors or in-transit due to time-activity patterns, indoor sources, and infiltration of outdoor sources into the home. MADRES is a well-characterized cohort, with a vast array of individual-level covariate data available, making this an excellent study for this research question to be conducted. Furthermore, this study provided evidence for the effect of PM_2.5_ sources on birthweight in a health disparities population, which may experience not just a greater burden of adverse health outcomes and environmental exposures, but also lower access to health care and resources to alleviate the impact of such burdens^68,69^.

The sample size of this study is a potential limitation with a final working sample of 198 (201 in the model that included the outliers), which while small for population-based health studies, is actually fairly large for personal exposure monitoring studies^70–72^. However, even with this potential limitation, we were able to detect several associations between major sources of PM_2.5_ and their respective high-loading components. Another possibility is participation bias due to differences in the type of expecting mothers that chose to participate in the personal exposure monitoring study component of the MADRES cohort study. However, we did not observe any material differences between participants who chose to participate in the personal exposure monitoring study compared to the larger cohort, except that they were slightly more likely to have had a prior child (data not shown).

A potential limitation of our study pertains to the techniques employed to analyze the mixtures of PM_2.5_ components and sources. While alternative methodologies were considered, we opted for the methods detailed in this paper for two main reasons. Firstly, given the constraints in sample size, we believed that venturing into other methodologies might not yield robust results. Secondly, our chosen source apportionment approach inherently serves as a form of mixture modeling, aligning with our research objectives, which primarily was to investigate the impact of PM_2.5_ sources, with the secondary aim of evaluating whether it was the source or specific components that drove the adverse health effect.

Finally, the 48-h sampling period in the 3rd trimester is a limitation as this may not be representative of typical or longer duration. The enhanced personal monitoring methods over shorter durations offer a more accurate estimation of genuine or “true” personal exposure compared to conventional modeled ambient techniques. However, there is a trade-off as they struggle to capture temporal variations due to short measurement times, which is especially concerning when representing extended periods like a pregnancy trimester. The shorter measurement period is unfortunately a limitation of the technology, with battery life and the burden to participants prohibitive for much longer time periods. Yet, the correlation between personal PM_2.5_ and ambient PM_2.5_ for the same 48-h sampling period was very similar to that with the 3rd trimester average ambient PM_2.5_^32^, suggesting the ambient segment of total PM_2.5_ exposure was likely consistent across the period. Also, we found reasonable concordance between time-activity patterns between two different questionnaire sources, including an exit survey after the 48-h sampling period, and a 3rd trimester questionnaire (data not shown), suggesting these exposure factors may not drastically differ. Together, these increase confidence in the representativeness of the measurements in terms of what participants are truly experiencing considering time-activity patterns and outdoor exposures are major contributing factors to personal PM_2.5_.

## Conclusion

Overall, this study found evidence that major outdoor sources of personal PM_2.5_ including fresh sea salt, aged sea salt, and to a lesser extent, SHS and crustal sources, were negatively associated with birthweight in a health disparities population in Los Angeles, CA. Mg, Na, Cl components were most strongly associated with negative birthweight. Additionally, the effect of crustal and fuel oil sources differed by infant sex with negative associations seen in boys compared to positive associations in girls.

### Supplementary Information

Supplementary Tables.

## Data Availability

The datasets used and/or analyzed during the current study are available from the corresponding author on reasonable request.
