# Supplementary material for: Effects of in-utero personal exposure to PM2.5 sources and components on birthweight
Source: Sci Rep. 2023 Dec 11;13:21987. doi: 10.1038/s41598-023-48920-w (PMC10713978; doi:10.1038/s41598-023-48920-w)
Supplement: Supplementary file 1 — Supplementary Tables. [file 41598_2023_48920_MOESM1_ESM.docx]

**SUPPLEMENTARY MATERIALS**

**Effects of In-Utero Personal Exposure to PM_2.5_ Sources and Components on Birthweight**

Karl O’Sharkey^1^; Yan Xu^2^; Jane Cabison^1^; Marisela Rosales^1^; Tingyu Yang^1^; Thomas Chavez^1^; Mark Johnson^1^; Deborah Lerner^4^, Nathana Lurvey^4^, Claudia M. Toledo Corral^1,3^; Shohreh F. Farzan^1^; Theresa M. Bastain^1^; Carrie V. Breton^1^; Rima Habre^1,2^

Institutions:

^1^Department of Population and Public Health Sciences, University of Southern California, Los Angeles, CA.

^2^Spatial Sciences Institute, University of Southern California, Los Angeles, CA.

^3^Department of Health Sciences, California State University Northridge, Northridge, CA.

^4^Eisner Health, Los Angeles, CA.

**Table S.1.** Personal PM_2.5_ Sources by Key Sample Demographics (N = 201).

|  | Birthweight | PM_2.5_ Mass | Traffic | SHS | Aged Sea Salt | Fresh Sea Salt | Fuel Oil | Crustal | |
| --- | --- | --- | --- | --- | --- | --- | --- | --- | --- |
|  | Spearman Correlation Coefficient | | | | | | | | |
| Maternal age (years) | 0.03 | 0.02 | -0.10 | -0.02 | 0.00 | 0.01 | -0.04 | 0.10 | |
| Gestational Age (weeks) | 0.43* | -0.06 | 0.19* | -0.09 | -0.02 | -0.02 | 0.05 | 0.08 | |
| Temperature (°C) | 0.01 | -0.12 | -0.09 | -0.07 | 0.65* | 0.03 | -0.12 | -0.11 | |
|  | Mean (SD)^b^ | | | | | | | | |
| Sex |  |  |  |  |  |  |  |  | |
| Female | 3,276.9 | 21.1 | 0.4 | 12.1 | 0.9 | 0.7 | 2.0 | 2.2 | |
| Male | 3,315.6 | 21.6 | 0.5 | 11.5 | 0.9 | 1.0 | 2.1 | 2.4 | |
| Race/ethnicity |  |  | * |  |  |  |  |  | |
| Hispanic | 3,293.8 | 21.6 | 0.5 | 12.0 | 1.0 | 0.9 | 2.1 | 2.3 | |
| Black, Non-Hispanic | 3,226.6 | 22.0 | 0.4 | 11.2 | 0.7 | 0.8 | 2.2 | 2.4 | |
| Other, Non-Hispanic | 3,410.6 | 17.4 | 0.2 | 10.6 | 0.5 | 0.5 | 1.9 | 1.3 | |
| Education |  |  |  |  |  |  |  |  | |
| <12th grade | 3,346.9 | 23.8 | 0.5 | 11.5 | 0.8 | 0.6 | 1.8 | 3.3 | |
| Completed High School | 3,208.6 | 21.6 | 0.4 | 12.5 | 1.0 | 1.0 | 2.2 | 2.1 | |
| Some college+ | 3,332.3 | 19.8 | 0.5 | 11.6 | 0.8 | 0.8 | 2.1 | 1.8 | |
| Diabetes |  |  |  | * |  |  |  |  | |
| Normal | 3,257.2 | 20.2 | 0.5 | 10.6 | 0.9 | 0.7 | 2.0 | 2.3 | |
| Glucose Intolerant | 3,339.2 | 22.9 | 0.4 | 15.2 | 0.9 | 1.2 | 2.1 | 1.7 | |
| Diabetes^ | 3,433.7 | 25.0 | 0.4 | 12.2 | 0.7 | 1.1 | 2.2 | 2.8 | |
| Pre-pregnancy BMI (kg/m^2^) |  |  |  |  |  |  |  |  | |
| Normal | 3,208.1 | 20.2 | 0.5 | 11.6 | 0.9 | 0.7 | 2.0 | 2.3 | |
| Overweight | 3,333.9 | 21.4 | 0.4 | 12.6 | 0.9 | 0.9 | 1.8 | 2.0 | |
| Obese | 3,334.1 | 22.1 | 0.4 | 11.4 | 0.8 | 0.9 | 2.3 | 2.4 | |
| Parity |  |  |  |  |  |  |  |  | |
| No | 3,179.8 | 18.6 | 0.4 | 11.2 | 0.8 | 0.6 | 2.3 | 1.6 | |
| Yes | 3,346.7 | 22.8 | 0.5 | 12.1 | 0.9 | 1.0 | 2.0 | 2.6 | |
| Missing | 3,531.7 | 21.3 | 0.4 | 12.7 | 1.5 | 0.5 | 1.2 | 1.7 | |
| Maternal Income |  |  |  |  |  |  |  |  | |
| <$15,000 | 3,314.6 | 25.6 | 0.5 | 13.3 | 1.0 | 0.8 | 2.1 | 2.6 | |
| $15,000-$29,999 | 3,335.8 | 19.6 | 0.5 | 10.5 | 0.9 | 0.8 | 2.3 | 1.6 | |
| $30,000+ | 3,346.8 | 19.9 | 0.5 | 9.6 | 0.9 | 1.2 | 2.0 | 2.9 | |
| Don't know | 3,231.2 | 20.8 | 0.4 | 13.2 | 0.8 | 0.7 | 1.9 | 2.1 | |
| Smoking History |  |  |  |  |  |  |  |  | |
| Never | 3,308.3 | 21.2 | 0.5 | 11.8 | 0.9 | 0.7 | 2.0 | 2.3 | |
| Ever | 3,247.1 | 22.0 | 0.5 | 12.1 | 0.8 | 1.4 | 2.1 | 2.3 | |
| Notes: Spearman correlation coefficients^a^ and Kruskal-Wallis one-way analysis of variance^b^; * = < 0.05; PM_2.5_ = particulate matter with an aerodynamic diabetes less than 2.5µm; SHS = secondhand smoke; test results were without missing or don't know level. | | | | | | | | |  |

**Table S.2**. Spearman’s Correlation Coefficients for High-Loading Components of the Six Personal PM_2.5_ Sources (N = 201).

| **Table S.3.** Effect Estimates (95% CI) of Major Personal PM_2.5_ Sources on Birthweight in Full Term Births Only. | | | | | | | | | | | | |
| --- | --- | --- | --- | --- | --- | --- | --- | --- | --- | --- | --- | --- |
|  | **Main Model (N = 180)** | | | | | | **Outliers Included (N = 183)** | | | | | |
| **Model** | **a** | | | **b** | | | **c** | | | **d** | | |
|  | No GA adjustment | | | Adjusted for GA | | | No GA adjustment | | | Adjusted for GA | | |
| Traffic | 6.7 | -63.9 | 77.2 | 48.7 | -26.7 | 124.1 | 11.0 | -62.2 | 84.2 | 55.0 | -23.2 | 133.2 |
| Secondhand Smoke | -3.6 | -62.3 | 55.1 | -23.3 | -87.2 | 40.5 | -11.8 | -72.1 | 48.5 | -33.7 | -99.2 | 31.8 |
| Aged Sea Salt | -70.4 | -146.9 | 6.2 | -36.2 | -120.0 | 47.6 | -46.1 | -119.8 | 27.7 | -11.6 | -92.0 | 68.8 |
| Fresh Sea Salt | -92.0 | -196.2 | 12.2 | -114.0 | -227.7 | -0.2 | 21.8 | -37.7 | 81.3 | 24.0 | -41.3 | 89.2 |
| Fuel Oil | 16.5 | -41.2 | 74.1 | 19.2 | -44.0 | 82.5 | 28.0 | -31.4 | 87.3 | 31.3 | -33.8 | 96.4 |
| Crustal | -16.2 | -106.4 | 74.0 | -18.7 | -117.7 | 80.2 | 74.3 | 16.4 | 132.1 | 80.4 | 16.9 | 143.9 |
| Notes: CI = confidence intervals; PM_2.5_ = particulate matter with an aerodynamic diabetes less than 2.5µm; β = change in birthweight per 1 SD increase in pollutant.  Models:  a) Full model in full-term births only and gestational age removed from the model as a covariate.  b) Full model in full-term births only and gestational age kept in the model as a covariate.  c) Full model with outliers included in full-term births only and gestational age removed from the model as a covariate  d) Full model with outliers included in full-term births only and gestational age kept in the model as a covariate. | | | | | | | | | | | | |
